# Cognitive Inflexibility in Gamblers is Primarily Present in Reward-Related Decision Making

**DOI:** 10.3389/fnhum.2014.00569

**Published:** 2014-08-13

**Authors:** Michiel Boog, Paul Höppener, Ben J. M. v. d. Wetering, Anna E. Goudriaan, Matthijs C. Boog, Ingmar H. A. Franken

**Affiliations:** ^1^Bouman Mental Health Care, Rotterdam, Netherlands; ^2^Institute of Psychology, Erasmus University Rotterdam, Rotterdam, Netherlands; ^3^Reinier van Arkel Groep, ‘s-Hertogenbosch, Netherlands; ^4^Department of Psychiatry, Academic Medical Center, Amsterdam Institute for Addiction Research, University of Amsterdam, Amsterdam, Netherlands; ^5^Arkin Mental Health Care, Amsterdam, Netherlands; ^6^Department of Public Health, Erasmus MC, Rotterdam, Netherlands

**Keywords:** gambling disorder, reward-based, cognitive inflexibility, reversal learning, WCST

## Abstract

One hallmark of gambling disorder (GD) is the observation that gamblers have problems stopping their gambling behavior once it is initiated. On a neuropsychological level, it has been hypothesized that this is the result of a cognitive inflexibility. The present study investigated cognitive inflexibility in patients with GD using a task involving cognitive inflexibility with a reward element (i.e., reversal learning) and a task measuring general cognitive inflexibility without such a component (i.e., response perseveration). For this purpose, scores of a reward-based reversal learning task (probabilistic reversal learning task) and the Wisconsin card sorting task were compared between a group of treatment seeking patients with GD and a gender and age matched control group. The results show that pathological gamblers have impaired performance on the neurocognitive task measuring reward-based cognitive inflexibility. However, no difference between the groups is observed regarding non-reward-based cognitive inflexibility. This suggests that cognitive inflexibility in GD is the result of an aberrant reward-based learning, and not based on a more general problem with cognitive flexibility. The pattern of observed problems is suggestive of a dysfunction of the orbitofrontal cortex, the ventrolateral prefrontal cortex, and the ventral regions of the striatum in gamblers. Relevance for the neurocognition of problematic gambling is discussed.

## Introduction

A gambling disorder (GD) is characterized by a lack of self-regulation (Goldstein et al., 2001; APA, 2013). Patients suffering from this disorder are not able to inhibit their urge to gamble, and are unable to shift their behavior (Goudriaan et al., 2008). Because of the similarities between GD and substance use disorders it is now generally seen as a non-substance related addictive disorder (APA, 2013). Further, there is clear connection between GD and obsessive compulsive disorder (Blaszczynski, 1999). A core feature of these both disorders is the inability to stop repetitive detrimental behavior.

One specific process related to the lack of self-regulation suggested to underlie GD is cognitive inflexibility associated with reward learning. More specifically, we refer to a tendency to hold on to behavior that has been profitable before, but no longer leads to gain (Klanker et al., 2013). In patients with GD, this reward-based cognitive inflexibility presumably can be observed as some kind of continuous gambling even in the face of increasing losses. Reward-based cognitive inflexibility can be studied using the principles of reversal learning, which is dependent on the capacity to perform flexible behavior when stimulus-reward contingencies alter (Clark et al., 2004; Franken et al., 2008). For example, in a study using a reversal learning task, GD-patients performed worse than healthy controls (de Ruiter et al., 2008). Reward-based cognitive inflexibility, i.e., reversal learning, has been associated with the orbitofrontal cortex (Klanker et al., 2013), the ventral prefrontal cortex (Clark et al., 2004), the ventrolateral prefrontal cortex (de Ruiter et al., 2008) and is facilitated by dopaminergic activity in the ventral regions of the striatum (Clark et al., 2004; Klanker et al., 2013). The concept of reward-based cognitive inflexibility is also closely related to the concept of reward sensitivity (Boog et al., 2013) and the concept of impaired decision making under conflicting contingencies (Goudriaan et al., 2008).

Another feature related to the lack of self-regulation is arguably a more general, non-reward-based, cognitive inflexibility seen in GDs. This form of cognitive inflexibility is based on the functioning of different regions of the prefrontal cortex and the basal ganglia (Monchi et al., 2001): the mid-dorsolateral prefrontal cortex, a cortical basal ganglia loop, the posterior prefrontal cortex, and the putamen. Klanker et al. (2013) stress the importance of the lateral prefrontal cortex in this form of cognitive inflexibility. Several studies show that GD-patients have non-reward-based cognitive inflexibility and suffer from perseveration, mainly studied by using the Wisconsin card sorting task (WCST; Rugle and Melamed, 1993; Regard et al., 2003; Goudriaan et al., 2006; Odlaug et al., 2011). Contradictory findings are, however, reported by Cavedini et al. (2002). They found no differences between healthy controls and GD-patients on the WCST and the Weigl’s sorting test, another instrument to test cognitive flexibility. In a later study, Brand et al. (2005) found no deviations in non-reward-based cognitive flexibility in GD as well.

Cognitive inflexibility with and without reward in GD are believed to be independent of each other (Cavedini et al., 2002). This idea is further supported by findings in substance dependent individuals (Bechara and Damasio, 2002). Cavedini et al. (2002) excluded a possible interference of non-reward-based cognitive inflexibility in abnormal decision making (i.e., reward-based cognitive inflexibility) in the Iowa gambling task. In this task, subjects have to choose between possible short-term high gains, resulting in eventual losses, or short-term smaller gains, resulting in overall gain in the long run. In contrast, Brand et al. (2005), however, suggested that there is a relationship between cognitive inflexibility with and without reward in GD. They used the modified card sorting test to investigate non-reward-based cognitive inflexibility. The game of dice task was applied to measure cognitive inflexibility with reward, a test in which the rules regarding gains and losses are explicit, contrary to the frequently used Iowa gambling task (in which these rules are implicit). In other words, in the game of dice task subjects receive explicit instructions on their chances of winning and losing. Brand and colleagues found that GD was associated with cognitive inflexibility with reward, but not with inflexibility without reward. Importantly, they found a relationship between cognitive inflexibility with and without reward in GD.

In the present study, the relationship between cognitive inflexibility with and without reward in GD was further investigated. It remains unclear whether basic cognitive inflexibility might play a role in cognitive flexibility that is based on rewards. The present study intended to clarify the nature of the presumed relationship between GD, reward-based cognitive inflexibility and non-reward-based cognitive inflexibility. Possibly, the difficulties with stopping detrimental behavior observed in GD is more a general problem of cognitive flexibility (i.e., behavioral perseveration as seen in obsessive compulsive disorders), and not only a reward-based decision making problem. It was hypothesized that GD-patients have higher levels of cognitive inflexibility without reward, that GD-patients are more cognitive inflexible with reward and that cognitive inflexibility with and without reward are not related. Further, the relationships between self-reported symptoms related to cognitive inflexibility (i.e., obsessive compulsive disorder symptoms), psychological distress, and cognitive inflexibility with and without reward were studied. This was done in order to find out if cognitive inflexibility was related to OCD-symptoms and psychological distress and to possibly obtain insight in which form of cognitive inflexibility is more related to and more relevant for the phenotypical manifestations of GD. Lastly, we studied the association between the cognitive inflexibility with and without reward in both GD and controls.

## Materials and Methods

### Participants

Thirty-eight individuals (male and female) participated in this study: 19 patients diagnosed with GD and 19 healthy controls. The GD-group consisted of outpatients of a large urban mental health care facility (Bouman Mental Health Care, Rotterdam, The Netherlands). The groups were matched regarding to gender and age. Characteristics of the two groups are presented in Table 1. GD-patients suffering from severe concomitant psychiatric disorders such as psychotic disorders, bipolar disorders, autism spectrum disorders, and neuropsychiatric disorders (as assessed by clinicians) and suffering from color blindness were not included. The healthy controls did not suffer from any psychiatric disorders or color blindness.

**Table 1:** **Characteristics of subjects**.

	GD-group	Healthy controls
Gender	14 Males, 5 females	16 Males, 3 females
Age	M = 42.1	M = 38.8
	SD = 13.35	SD = 8.0
Level of education	1 = 26.3%	1 = 42.1%
	2 = 52.6%	2 = 52.6%
	3 = 15.8%	3 = 0%
	4 = 5.3%	4 = 5.3%
Years of education	M = 13.47	M = 15.11
	SD = 4.0	SD = 2.47

*Level of education: 1 = high, 2 = intermediate, 3 = low, 4 = no education*.

*M, mean; SD, standard deviation*.

### Procedure

Individuals who were included in the GD-group were all members of a standard group therapy for GD. Potential participants were informed about the procedure and when they were willing to take part, they signed an informed consent form. Matched controls were selected via convenience sampling. The study was approved by the Ethics Commission of the Reinier van Arkel Groep. After participants agreed to participate in the study, the behavioral tests were administered, questionnaires were filled out and personal information was acquired. The data collection took place in the treatment facility.

### Instruments

The probabilistic reversal learning task (PRLT) was used as a measure of cognitive inflexibility (Clark et al., 2004; Franken et al., 2008). The PRLT measures reward-based response perseveration. In this study, we used the version of the PRLT as described by Franken et al. (2008). During 100 trials, subjects had to choose between two stimuli (S^+^ and S^−^, easily discernable geometrical figures), presented on a computer screen. The S^+^ (advantageous) stimulus had the following properties: a reward–punishment ratio of 70:30, a reward range of 80–250 points, and a punishment range of 10–60 points. For the S^−^ (disadvantageous) stimulus the reward–punishment ratio was 40:60, the reward range 30–60 points, and the punishment range 250–600 points. The continuous choice of S^+^ lead to overall gain, the continuous choice of S^−^ resulted in overall loss. At onset, subjects did not know which geometrical figure resulted in overall gain and which in overall loss. They were supposed to learn this by trial and error. A reversal took place after five correct (S^+^) choices. Then the geometrical figure leading to the advantageous outcome became the geometrical figure leading to the disadvantageous outcome and vice versa. The total number of reversals (i.e., the capacity to change strategy) was the outcome variable of interest (PRLT reversals).

The WCST (Heaton, 1981) was used to investigate a more general, non-reward-based, cognitive inflexibility, which is reflected in the number of response perseverations (Goudriaan et al., 2006). In this test, subjects have to sort cards in such a way that they match one of four stimulus cards, according to a concept that is unknown to the subject (form, color, or number). Feedback is provided regarding the correctness of the response. After 10 consecutive correct responses the sorting principle changes and the subject has to change strategy. We used percentage of perseverative responses as variable in our analyses (WCST perseverations).

The brief symptom inventory (Derogatis and Melisaratos, 1983) is a self-report measure, derived from the SCL-90-R (Derogatis et al., 1976). It is a brief psychological symptom scale. The Dutch version of the BSI has solid validity and reliability (De Beurs and Zitman, 2005), with a Cronbach’s alpha of 0.96. Although the BSI comprises nine subscales, the total score was used as index of current distress in this study.

The Padua inventory (PI; Sanavio, 1988) is an obsessive compulsive disorder symptom questionnaire. In a sample of Dutch subjects, the validity and reliability of the PI were satisfactory (van Oppen, 1992), with a Cronbach’s alpha of 0.94. In the present study, the Padua Inventory-Revised (PI-R) was used (Van Oppen et al., 1995; Anholt et al., 2009). It has five subscales: impulses, washing, checking, rumination, and precision. Further, it yields a total score, indicating severity of OCD-symptoms. Some evidence exists for the construct validity of the PI-R. (Van Oppen et al., 1995).

The South Oaks Gambling Screen (SOGS; Lesieur and Blumen, 1987; Stinchfield, 2002) is a short screening instrument for pathological gambling, based on DSM criteria. It has adequate psychometric qualities (Lesieur and Blumen, 1987), with a Cronbach’s alpha of 0.97. In the present study, a Dutch version of the SOGS was used (Goudriaan, 2013). The SOGS was used as a measure of severity of GD.

### Data analysis

Analyses were conducted to investigate possible differences between GD-patients and controls regarding age, education, and gender. Education was operationalized as level of education and years of education. Level of education was divided in four categories: high (college/university), intermediate (high school), low (junior high school), and no secondary education. An independent-samples *t*-test was used to investigate age differences and years of education, a Fisher’s exact test was used for gender (because both variables in this analysis were categorical, and because of small sample size). A Mann–Whitney *U*-test was executed to compare the two groups regarding level of education, because level of education is an ordinal variable. Further, independent-samples *t*-tests were used to compare the group of GD-patients with normal controls regarding scores on PRLT, WCST, PI-R, and BSI. To compare both groups regarding scores on the SOGS, a Mann–Whitney *U*-test was employed, because of non-normality of the distribution of the values of this variable. In order to study relationships between BSI, PI-R, PRLT, and WCST correlations between these variables were determined. These correlations were computed within the GD-group, in the control group and in the total group (GD-patients and healthy controls together). All tests were done two-tailed with an alpha level of 0.05.

## Results

### Demographics

An independent-samples *t*-test was conducted to compare the age of patients with GD and controls. No significant difference was found in age between GD-patients (M = 42.05, SD = 13.35) and controls (M = 38.79, SD = 7.79): *t*(29.39) = 0.92, *p* = 0.37. Another independent-samples *t*-test was conducted to find out if the two groups differed regarding years of education. No significant difference was found in years of education between GD-patients (M = 13.47, SD = 4.01) and controls (M = 15.11, SD = 2.47): *t*(36) = −1.51, *p* = 0.14 (two-tailed). A significant difference between level of education of GD-patients and controls was, however, found (*p* = 0.025) using a Mann–Whitney *U*-test. Of the patients with GD, 26.3% had a low educational level versus 0% of the controls; 42.1% of the controls had a high educational level versus 15.8% in the control group. The control group had a higher level of education. For gender, Fisher’s exact test was employed. No significant differences in gender were found (*p* = 0.69).

### Differences between groups regarding reward-based cognitive inflexibility, non-reward-based cognitive inflexibility, severity of OCD-symptoms, psychological distress, and severity of GD

Mean scores (SD) of all five measures (PRLT reversals, WCST perseverations, BSI, PI-R, and SOGS) are displayed in Table 2. To find out if level of education was a possible confounder, Spearman rank order correlations between level of education and the several dependent variables were calculated as a pre-test. No significant correlations were observed (correlation between educational level and WCST perseveration was 0.14, and between education and PRLT reversals was −0.02).

**Table 2:** **Mean scores (SDs) of GD-patients, healthy controls, and total group on PRLT reversals, WCST perseverations, BSI, PI-R, and SOGS**.

	GD-group	Healthy controls	Total group
PRLT reversals	4.1 (2.2)	5.9 (2.7)	5.0 (2.6)
WCST perseverations	18.9 (11.4)	12.5 (5.9)	15.7 (9.5)
BSI	1.1 (1.1)	0.14 (0.12)	0.6 (0.9)
PI-R	36.1 (23.2)	18.9 (11.5)	27.5 (20.1)
SOGS	8.3 (3.4)	0.21 (0.71)	4.2 (4.7)

Independent-samples *t*-tests (PI-R, BSI, PRLT reversals, and WCST perseverations) and a Mann–Whitney *U*-test (SOGS) were conducted to compare the GD-group and the control group. As expected, GD-patients reported significantly higher scores on the SOGS (*p* < 0.001) than controls. Also, GD-patients had higher scores on the PI-R total score [*t*(26.32) = 2.90, *p* = 0.01]. On only two subscales of PI, patients with GD obtained significantly higher scores (rumination and precision). The two groups also differed regarding the BSI [*t*(18.48) = 3.76, *p* = 0.001], GD-patients obtaining higher scores. Further, on the PRLT, GD-patients reached a lower number of reversals [*t*(36) = −2.39, *p* = 0.022]. No significant difference was found between the two groups regarding percentage of perseverative responses in the WCST [*t*(36) = 1.07, *p* = 0.29].

### Correlations between reward-based cognitive inflexibility, non-reward-based cognitive inflexibility, psychological distress, and severity of OCD-symptoms

Inspection of scatterplots regarding the respective relationships between the BSI, PI-R, WCST perseverations, and PRLT reversals, neither revealed any outliers nor deviations from normality.

Correlations were computed between PI-R, BSI, PRLT reversals, and WCST perseverations in the GD-group, the control group and the total group. Results can be found in Tables 3–5. In GD-patients, significant correlations were found between PRLT reversals and BSI (higher number of reversals associated with lower BSI-scores), and between BSI and PI-R (higher BSI-scores co-occur with higher PI-R-scores). The correlation between PRLT reversals and PI-R only approached significance. More reversals were linked to lower PI-R-scores. In the control group, no significant correlations appeared. In the total group, significant correlations were found between PRLT reversals and BSI (more reversals associated with lower BSI-scores), between PRLT reversals and PI-R (more reversals associated with lower PI-R-scores), and between BSI and PI-R (higher BSI-scores co-occurred with higher scores on PI-R).

**Table 3:** **Correlations between several variables in the GD-group**.

	PRLT	WCST	PI-R	BSI
	reversals	perseverations		
PRLT reversals		−0.21	−0.45^a^	−0.52^b^
WCST perseverations			−0.02	0.14
PI-R				0.78^c^
BSI				

*^a^*p* = 0.054*.

*^b^Correlation is significant at the 0.05 level (two-tailed)*.

*^c^Correlation is significant at the 0.01 level (two-tailed)*.

**Table 4:** **Correlations between several variables in the control group**.

	PRLT	WCST	PI-R	BSI
	reversals	perseverations		
PRLT reversals		−0.19	−0.07	0.01
WCST perseverations			−0.25	0.05
PI-R				0.40
BSI				

**Table 5:** **Correlations between several variables in the total group (GD and control)**.

	PRLT	WCST	PI-R	BSI
	reversals	perseverations		
PRLT reversals		−0.24	−0.39^a^	−0.45^b^
WCST perseverations			0.01	0.19
PI-R				0.78^b^
BSI				

*^a^Correlation is significant at the 0.05 level (two-tailed)*.

*^b^Correlation is significant at the 0.001 level (two-tailed)*.

## Discussion

The present study investigated the nature of the presumed relationship between GD and cognitive inflexibility. Significant differences were found between a group of patients suffering from GD and healthy controls (matched regarding age and gender). GD-patients reported higher levels of severity of gambling, higher levels of psychological distress, and more obsessive compulsive symptoms. Further, they displayed more reward-based cognitive inflexibility. No evidence was found that GD was related to non-reward-based cognitive inflexibility, i.e., perseveration. In the GD-group, reward-based and non-reward-based cognitive inflexibility were not related. Reward-based cognitive inflexibility was significantly related to level of psychological distress; more inflexible GD-patients reporting more symptoms. A near-significant correlation was found between reward-based cognitive inflexibility and OCD-symptoms. Non-reward-based cognitive inflexibility was not related to level of psychological distress or OCD-symptoms.

In the control group, no relationship was found between the two forms of cognitive inflexibility, and between cognitive inflexibility and symptoms. In the total group, however, significant relationships were found between reward-based cognitive inflexibility on one side and psychological distress and OCD-symptoms on the other. Non-reward-based cognitive inflexibility was not related to level of psychological distress or OCD-symptoms. Once again, reward-based and non-reward-based cognitive inflexibility were not related.

The findings in the present study suggest that reward-based cognitive inflexibility characterizes GD-patients in contrast to non-reward-based cognitive inflexibility. GD-patients, in other words, do not seem to have problems with general flexibility in strategy and behavior, but do have difficulties with altering a response that is rewarded before, but no longer is (i.e., a dysfunctional focus on rewards). This is line with findings of several studies that suggest that GD is intertwined with reward-based cognitive inflexibility (Cavedini et al., 2002; Brand et al., 2005; de Ruiter et al., 2008). The idea that GD is not related to general flexibility is supported by two studies (Cavedini et al., 2002; Brand et al., 2005), but is contradicted by results from other studies (Rugle and Melamed, 1993; Regard et al., 2003; Goudriaan et al., 2006; Odlaug et al., 2011). Also, the present study found indications that reward-based and non-reward-based cognitive inflexibility are not related in GD. Therefore, it seems that the reward-based cognitive inflexibility observed in GD is not the result of a more general, non-reward-based tendency to perseverate. The results of different studies support these findings (Bechara and Damasio, 2002; Cavedini et al., 2002). Brand et al. (2005), however, suggest that non-reward-based cognitive inflexibility in itself is not a characteristic of GD [which is supported by the findings of Cavedini et al. (2002)], but plays a role in reward-based cognitive inflexibility, when the rules about winning and losing are explicit.

The present study gives preliminary evidence for the idea that reward-based cognitive inflexibility is a more central feature of GD than non-reward-based cognitive inflexibility. This conception is further supported by the finding that in the GD-group reward-based inflexibility was related to level of psychological distress and nearly significantly related to level of OCD-symptoms; in the total group reward-based cognitive inflexibility was related to psychological distress and OCD-symptoms. In other words, subjects who were inflexible when rewards were at stake, were more obsessive and compulsive and reported lower levels of psychological well-being. Cognitive inflexibility without reward was not related to OCD-symptoms and psychological distress. This is another indication that GD might be more a problem of reward-based inflexibility than of a general tendency to perseverate. This stresses the importance of possible dysfunctioning of the orbitofrontal cortex, the ventrolateral prefrontal cortex, and the ventral regions of the striatum in GD-patients (Clark et al., 2004; Klanker et al., 2013).

Some limitations of this study should be mentioned. Firstly, the size of the sample was relatively small. Further, the rules for winning and losing in the PRLT are implicit. That leaves open the possibility that non-reward-based cognitive inflexibility plays a role in reward-based cognitive inflexibility (see Brand et al., 2005).

In this study, the relations between reward-based and non-reward-based cognitive inflexibility and GD were investigated. In the context of comparing non-reward-based and reward-based cognitive inflexibility in GD, the paradigm of reversal learning is used for the first time. It is likely that reward-based cognitive inflexibility is a more central aspect of GD.

## Author Contributors

Paul Höppener, Ingmar H. A. Franken, Ben J. M. v. d. Wetering, and Anna E. Goudriaan designed the study. Paul Höppener collected the data. Michiel Boog and Ingmar H. A. Franken conducted the statistical analyses. Michiel Boog and Matthijs C. Boog wrote the first draft of the manuscript and all authors contributed to and have approved the final manuscript and agree to be accountable for all aspects of the work.

## Conflict of Interest Statement

The Guest Associate Editor Ali Mazaheri declares that, despite being affiliated to the same institution as author Anna E. Goudriaan, the review process was handled objectively and no conflict of interest exists. The authors declare that the research was conducted in the absence of any commercial or financial relationships that could be construed as a potential conflict of interest.
